# APPLYING A BILL OF RIGHTS FOR PEOPLE UNDER GUARDIANSHIP IN RURAL AREAS

**DOI:** 10.1093/geroni/igad104.2884

**Published:** 2023-12-21

**Authors:** Pamela Teaster, E Carlisle Shealy

**Affiliations:** Virginia Tech, Blacksburg, Virginia, United States; Virginia Tech, Blacksburg, Virginia, United States

## Abstract

In the U.S., 35.2% of people aged 65 and older live with a disability, of which 8.9% have a cognitive disability. For some, cognitive disabilities necessitate the help of a surrogate decision-maker (e.g., a guardian), including adults with serious mental illness, intellectual disability, and traumatic brain injury. Guardians are bound by statutory requirements, case law, and ethical principles to act in the best interests of an adult adjudicated by a court to lack the capacity to make certain decisions. Guardians are charged to act according to the highest standards of care, accountability, trust, honesty, confidentiality, and avoidance of conflict of interest. Powers given to guardians are often immense (e.g., authority to sell a person’s home and personal property, consent to medical treatments). Little is known about the characteristics of people with a guardian. Our study in rural Virginia examined all new guardianships of adults in one jurisdiction across three years (89% Caucasian, 54% male). Their most common disabilities were dementia and intellectual disabilities. No guardian’s report indicated any improvements in the adults’ mental or physical health. The National Guardianship Network, a collaboration of 13 national organizations, drafted a Bill of Rights for Adults with a Guardian, categorizing rights always retained after a guardian is appointed, personal rights that a court may restrict but not delegate to a guardian, and rights a guardian may exercise on behalf of an adult. Characteristics of a population of persons with a guardian living in a rural area are presented alongside considerations of their rights.

